# Chemical Profiling and In Vitro Bioactivities of Extracts from the Red Alga *Jania rubens* Collected from the Lebanese Coast

**DOI:** 10.3390/md24090295

**Published:** 2026-08-24

**Authors:** Rayan Kassir, Fatima El-Mched, Zeina Radwan, Zeina Dassouki, Hiba Mawlawi

**Affiliations:** 1 Laboratory of Applied Biotechnology (LBA3B), AZM Center for Research in Biotechnology and Its Applications, Doctoral School for Sciences and Technology, Lebanese University, Tripoli 1300, Lebanon; rayan.alkassir@ul.edu.lb; 2 Department of Biological Science, Faculty of Science, Beirut Arab University, Tripoli P.O. Box 11-5020, Lebanon; fatima.mashad@bau.edu.lb; 3 Department of Anatomy, Cell Biology, and Physiological Sciences, Faculty of Medicine, American University of Beirut, Beirut 1107-2020, Lebanon; zar04@mail.aub.edu; 4 Department of Medical Laboratory Sciences, Faculty of Health Sciences, University of Balamand, Sin El Fil P.O. Box 55251, Lebanon; 5 Faculty of Public Health III, Lebanese University, Tripoli 1300, Lebanon

**Keywords:** *J. rubens*, α-amylase inhibition, anticoagulant activity, anti-inflammatory activity, anti-hemolytic activity, GC-MS, HPLC, bioactive compounds

## Abstract

*Jania rubens* (*J. rubens*), a Mediterranean red seaweed, is rich in bioactive compounds with potential medicinal applications. This study investigates the therapeutic potential of Lebanese *J. rubens* through in vitro evaluation of its anti-diabetic, anti-coagulant, anti-inflammatory and anti-hemolytic activities. Various extracts were prepared and characterized using GC-MS and HPLC. Biological activities were assessed through α-amylase inhibition assays, effects on prothrombin time and partial thromboplastin time, anti-inflammatory activity and anti-hemolytic activity. Significant α-amylase inhibition was observed with dichloromethane/methanol and lipid extracts, comparable to acarbose. All types of extracts prolonged PT and PTT, with the lipid extract showing the strongest effect at higher concentrations. Additionally, dichloromethane/methanol extracts exhibited potent anti-hemolytic activity. Moreover, all extracts showed strong anti-inflammatory activity, achieving levels of inhibition of heat-induced BSA denaturation comparable to diclofenac. Characterization revealed 11 amino acids in the protein extracts, and significant fatty acids in the lipid profile. The crude extracts contained diverse compounds, including flavonoids, aldehydes, diterpenoids, terpenoids, fatty acids, and sterol esters, which may contribute to the observed biological activities. These results highlight *J. rubens* as a potential reservoir of bioactive compounds with promising in vitro activities related to diabetes, thrombotic disorders, inflammation, and oxidative stress. Further research is needed to isolate the active compounds, validate their efficacy in vivo, assess safety, and elucidate the underlying mechanisms.

## 1. Introduction

For decades, natural products have been utilized in the treatment of various diseases due to their effectiveness, economic viability, and availability. There is growing interest in marine organisms, particularly algae, for therapeutic drug manufacturing [1]. Marine macroalgae, or seaweeds, are multicellular, photosynthetic eukaryotes categorized into three primary groups based on color: Rhodophyta (red), Phaeophyta (brown), and Chlorophyta (green) [2]. These macroalgae harbor a high percentage of water, carbohydrates, proteins, and low amount of lipids [3]. They are recognized for diverse species and high nutritional content and possess multiple bioactivities, such as antioxidant, antimicrobial, anti-obesity, anti-diabetic, anticancer, anti-inflammatory, antiallergic, cytotoxic, antiviral properties and anticoagulant effects [4,5,6].

Diabetes mellitus (DM), a major worldwide health issue, is a metabolic disorder marked by persistent high blood glucose levels that can result in severe complications [7]. A key contributor to elevated glucose levels is the enzymatic hydrolysis of carbohydrates by α-glucosidase and α-amylase [8]. Clinically approved inhibitors of these enzymes, such as Acarbose, Voglibose, and Miglitol, are often used alongside hypoglycemic drugs such as metformin for the treatment of diabetes to reduce HbA1c levels [9]. However, these drugs are expensive and often accompanied by severe side effects [10].

Thromboembolic disorders like heart attack, deep vein thrombosis, and pulmonary embolism are likewise leading contributors to mortality around the world, especially in developing countries [11]. Existing anticoagulant agents such as heparins and vitamin K-antagonists are extensively employed in clinical practice to prevent thrombosis, but they are costly and associated with potentially life-threatening adverse effects [12].

Moreover, Inflammation is the immune system’s standard physiological reaction to external stimuli such as pathogens, toxins, and cellular damage. This response may result in acute or chronic inflammatory reactions across different organs, leading to tissue injuries and immune diseases [13]. When inflammatory mediators are produced excessively or without proper regulation, they can amplify inflammation and injure healthy tissues, which may lead to autoimmune disorders [14]. Nonsteroidal anti-inflammatory drugs (NSAIDs), including ibuprofen, diclofenac, and aspirin, are effective conventional medications; however, prolonged use can produce harmful effects on the gastrointestinal tract, kidneys, and liver [15,16]. Natural products derived from different algal species, namely alkaloids, flavonoids, terpenoids, steroids, and phenols, have attracted considerable attention for their potential anticoagulant, and antidiabetic and anti-inflammatory properties [10,17,18].

On a different note, a critical consideration in drug development is the hemolytic potential of bioactive novel therapeutic agents. Hemolysis is the breakdown of red blood cells, causing their internal contents to enter the surrounding fluid. This process is predominantly triggered by oxidative damage to the erythrocyte membrane, which originates from various factors, including certain hemoglobinopathies, oxidizing medications, elevated levels of transition metals, ionizing radiation exposure, and impaired red blood cell antioxidant systems [19]. In this framework, the assessment of hemolytic activity constitutes a useful starting point, as the toxicity of bioactive molecules is a key factor in therapeutic design [20].

*Jania rubens* (*J. rubens*), a red alga belonging to the order Corallinales, is widely distributed in the Mediterranean and Black Seas, as well as the northeastern Atlantic and China Sea, and the Indian Ocean [21]. It has dichotomously branched, cylindrical thalli forming dense mats, and can grow both attached to substrates or as an epiphyte [22]. This alga has been extensively studied due to its wide range of biological activities [21,23]. Our research team in Lebanon has demonstrated that various *J. rubens* extracts exhibit antioxidant and anticancer activities against colorectal cancer cells [24,25]. In particular, MTT reduction and wound-healing assays revealed that organic extracts (DM crude and Soxhlet) significantly suppressed the proliferation of colon cancer cell lines (HCT-116 and HT-29) and inhibited their migratory and metastatic potential in a dose- and time-dependent manner [24]. Furthermore, all *J. rubens* extracts (lipids, proteins and polysaccharides) exhibited significant anticancer effects, with the polysaccharide extract showing particularly strong cytotoxicity, apoptosis induction, and antiproliferative and anti-migratory activities [25]. These findings align with previous studies reporting the anticancer activity of *J. rubens* extracts [22,26,27,28,29,30,31]. In addition, Lebanese Jania rubens sulfated polysaccharide extract showed dual in vitro activity: antibacterial effects against *Streptococcus agalactiae* and enhancement of doxorubicin cytotoxicity in colorectal cancer cell lines [32]. However, despite its broad distribution [33], few studies have evaluated different *J. rubens* extracts collected specifically from the Lebanese coast [24,25,32,34] and none have investigated multiple biological activities simultaneously. FTIR characterization of polysaccharide and GC-MS profiling of DM-Soxhlet extracts have been reported in our previous studies [33,34,35]. However, the present study extends the chemical characterization to the lipid, Protein, and DM crude extracts and evaluates potential relationships between the chemical constituents and observed biological effects. Therefore, the novelty of this research lies in the combined chemical characterization of multiple *J. rubens* extracts and comprehensive evaluation of several biological activities, including anti-diabetic, anticoagulant, anti-hemolytic, and anti-inflammatory effects.

## 2. Results

### 2.1. Characterization of J. rubens Protein Extract Using HPLC

The protein extract contained 11 amino acids (Table 1), including four essential amino acids (phenylalanine, leucine, valine, and methionine), six non-essential amino acids (glutamic acid, aspartic acid, tyrosine, alanine, glycine, and ornithine), and one conditionally essential amino acid (arginine). Quantitative analysis revealed that glutamic acid was the most abundant, accounting for 24.6% of the total amino acid content, followed by aspartic acid (18.2%) and leucine (12.4%). In contrast, glycine and alanine were present at lower levels (5.1% and 4.8%, respectively). These relative abundances provide insight into the nutritional and functional composition of *J. rubens* extracts. Product ion scan spectra further confirmed prominent fragments at *m*/*z* 158.1 for glutamic acid and 144.1 for aspartic acid, respectively.

### 2.2. Characterization of J. rubens Using GC-MS Analysis

#### 2.2.1. Lipid Profiling of *J. rubens*

GC-MS profiling of lipid extracts from *J. rubens* identified a diverse array of lipid compounds, comprising both fatty acids and other lipid derivatives. A total of 11 distinct compounds were detected, as listed in Table 2, each characterized by varying retention times, molecular masses, relative abundances, and chemical classes. Importantly, several saturated fatty acids were identified, including hexadecanoic acid (palmitic acid, RT: 48.879 min; *m*/*z*: 97.1), octadecanoic acid (stearic acid, RT: 46.015 min; *m*/*z*: 149), dodecanoic acid (lauric acid, RT: 20.065 min; *m*/*z*: 57), and eicosanoic acid (arachidic acid, RT: 29.138 min; *m*/*z*: 57).

Among the non-fatty acid constituents, 2,4-Di-tert-butylphenol (RT: 36.013 min; *m*/*z*: 191.1) exhibited the highest concentration, while other notable compounds included Neophytadiene (RT: 44.807 min; *m*/*z*: 105.1) and Phytol (RT: 52.038 min; *m*/*z*: 85.1). The identified lipid components and derivatives could have implications for nutrition, health, and industry, particularly in the formulation of dietary supplements, cosmetics, and pharmaceuticals.

#### 2.2.2. *J. rubens* DM-Crude Extract

GC-MS profiling of the DM-crude of *J. rubens* showed a mixture of diterpenoids, terpenoids, saturated fatty acids and sterol ester compounds (Table 3). A total of 100 peaks were observed, each corresponding to specific chemical constituents identified using the NIST 14 spectral database installed in the GC-MS. The main chemical constituents were: Neophytadiene (RT: 45.179 min), followed by 2-Pentadecanone,6,10,14-trimethyl- (RT: 45.349 min), Hexadecanoic acid (RT: 47.362 min), Phytol (RT: 51.604 min) and Cholesteryl 3-cyclohexylbutyrate (RT: 57.132 min). 

#### 2.2.3. *J. rubens* DM-Soxhlet Extract

In our previous study, GC-MS analysis of *J. rubens* DM-Soxhlet extract demonstrated the presence of numerous bioactive secondary metabolites. The predominant constituents included flavonoid derivatives such as 4′-Methoxyflavone, 7-Methoxyflavone, 4H-1-Benzopyran-4-one, 8-methoxy-2-phenyl- (commonly referred to as 8-methoxyflavone) and 2-[3-Methoxyphenyl]-4H-1-benzopyran-4-one, all of which showed strong library matching and comparatively large peak areas. Pyrazole-derived molecules were likewise detected, such as 5-amino-1,3-diphenyl-1H-pyrazole, 1-(Anilinomethylene)-2-indanone, and Indan-1,3-dione, 2-(1,3-dimethyl-1H-pyrazol-4-ylmethylene). In addition, several heterocyclic and nitrogen-containing constituents were identified, including 4-[5-(4-Methoxyphenyl)-2-oxazolyl] pyridine and Oxazolo [3,2-e] xanthine. Other detected constituents included 4-bromo-N-(2-chloroacetyl) pyrazole-1-carboxamide and 2,3-dihydro-2-hydroxymethyl-5,7-dimethyl-4H-1-benzopyran-4-one [34].

### 2.3. Polysaccharide FTIR Analysis

**Figure 1:**
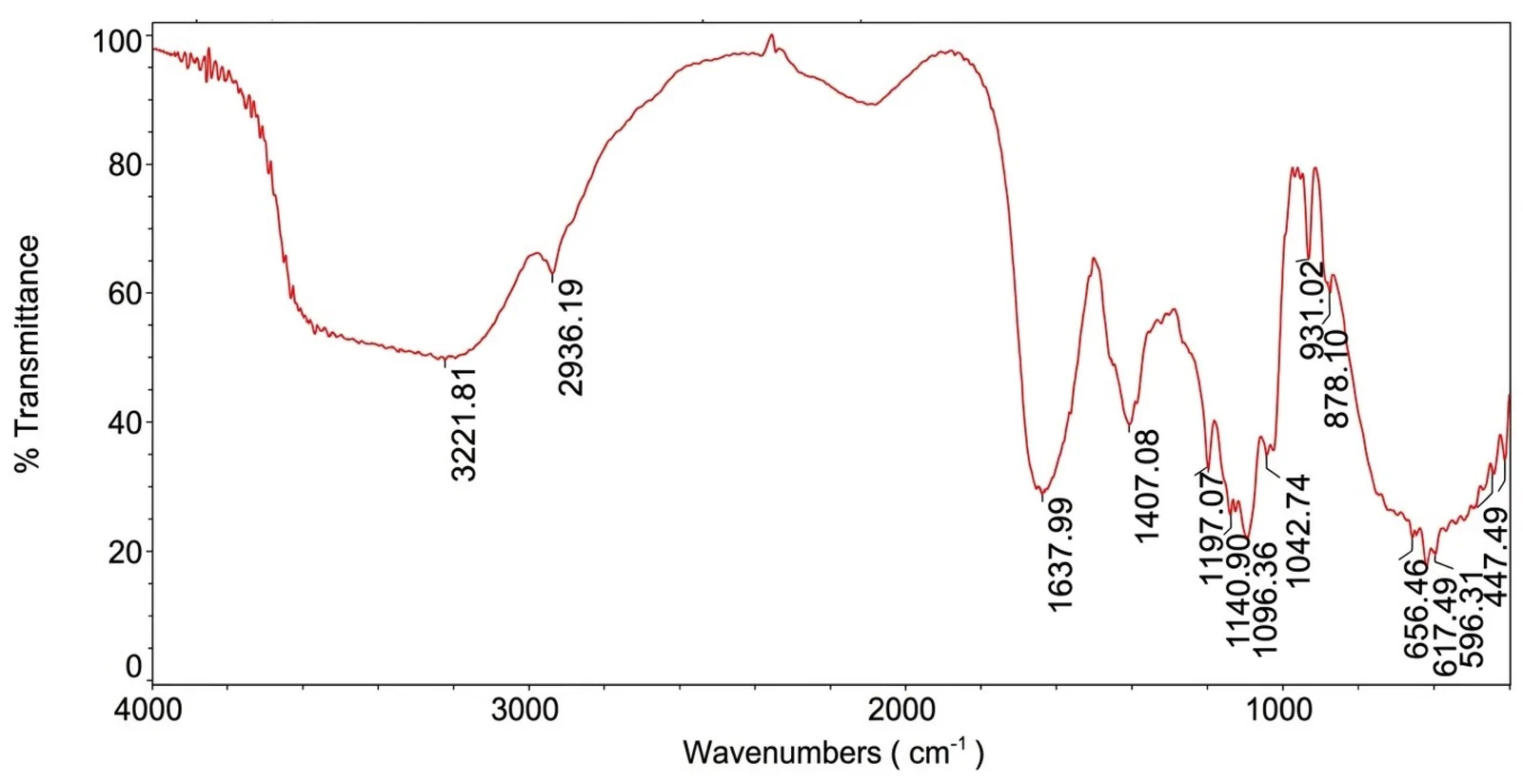
FT-IR analysis of polysaccharides extracted from the red algae *J. rubens*.

The FT-IR spectrum of the polysaccharides has been characterized previously by our team (Figure 1) [25]. Its spectral profile corresponded well with the characteristic FT-IR pattern of carrageenan, reported in earlier studies. More specifically, the broad absorption band observed between 3221 and 3545 cm^−1^ was assigned to the stretching mode of hydroxyl groups (OH). The band at 2936 cm^−1^ was associated with C–H stretching, a typical feature of sugar moieties. Additionally, the bands at 1197 cm^−1^ and 878 cm^−1^ suggested the presence of ester sulfate groups at the C4 position of galactose residues. The signals at 931 cm^−1^ and 1042 cm^−1^ were attributed to C-O-C stretching vibrations of 3,6-anhydrogalactose units.

### 2.4. Effect of J. rubens Extracts as Anti-Diabetic

**Figure 2:**
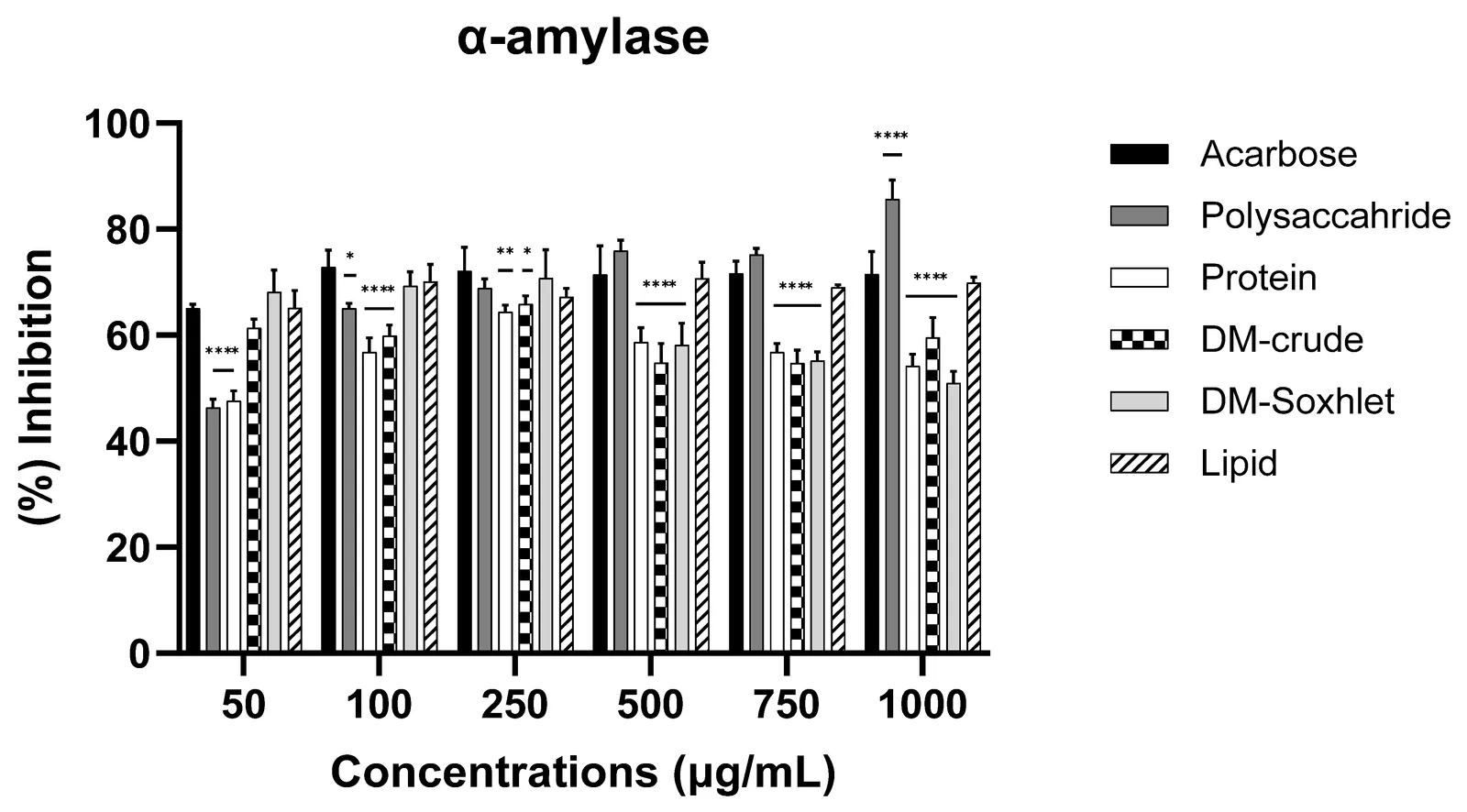
Percent inhibition of Alpha-amylase by various extracts and Acarbose as standard. Data are expressed as mean ± SD from triplicate experiments. Statistical significance was determined relative to the control, with *p* < 0.05 considered significant; * *p* < 0.05; ** *p* < 0.01; **** *p* < 0.0001. Error bars represent standard deviations.

Different extracts with varying concentrations exhibit distinct effects on alpha-amylase inhibition (*p* < 0.0001). The Dichloromethane Methanol Soxhlet extract (DM-Soxhlet) and the lipid extract exhibited strong inhibitory effects on α-amylase, comparable to those of acarbose at lower concentrations. At 50, 100, and 250 μg/mL, the lipid extract inhibited α-amylase activity by approximately 65%, 70%, and 67%, respectively, while the DM-Soxhlet extract produced inhibition values of 68%, 69%, and 79%, respectively. These activities were comparable to those of acarbos, which produced inhibition values of 65%, 72%, and 72% at the corresponding concentrations (*p* > 0.05) (Figure 2, Table 4). The lipid extract maintained a relatively stable inhibitory effect at higher concentrations (500–1000 μg/mL), showing inhibition values ranging from 65% to 72%, which were comparable to those observed for acarbose (69–71%) (*p* > 0.05). Although the DM-Soxhlet extract displayed a slight decrease in inhibition at higher concentrations, this pattern may reflect extract interference or compound interactions affecting enzyme kinetics. At high concentrations, the polysaccharide extract showed superior inhibition of α-amylase compared to acarbose. Specifically, at concentrations of 500, 750, and 1000 µg/mL, the polysaccharide achieved inhibition rates of 75.97%, 75.21%, and 85.73%, respectively, while acarbose yielded inhibition rates of 71.47%, 71.59%, and 71.57% (Figure 2, Table 4).

### 2.5. Effects of Various Extracts on Prothrombin Time and Partial Thromboplastin Time

#### 2.5.1. Effect on Prothrombin Time

**Figure 3:**
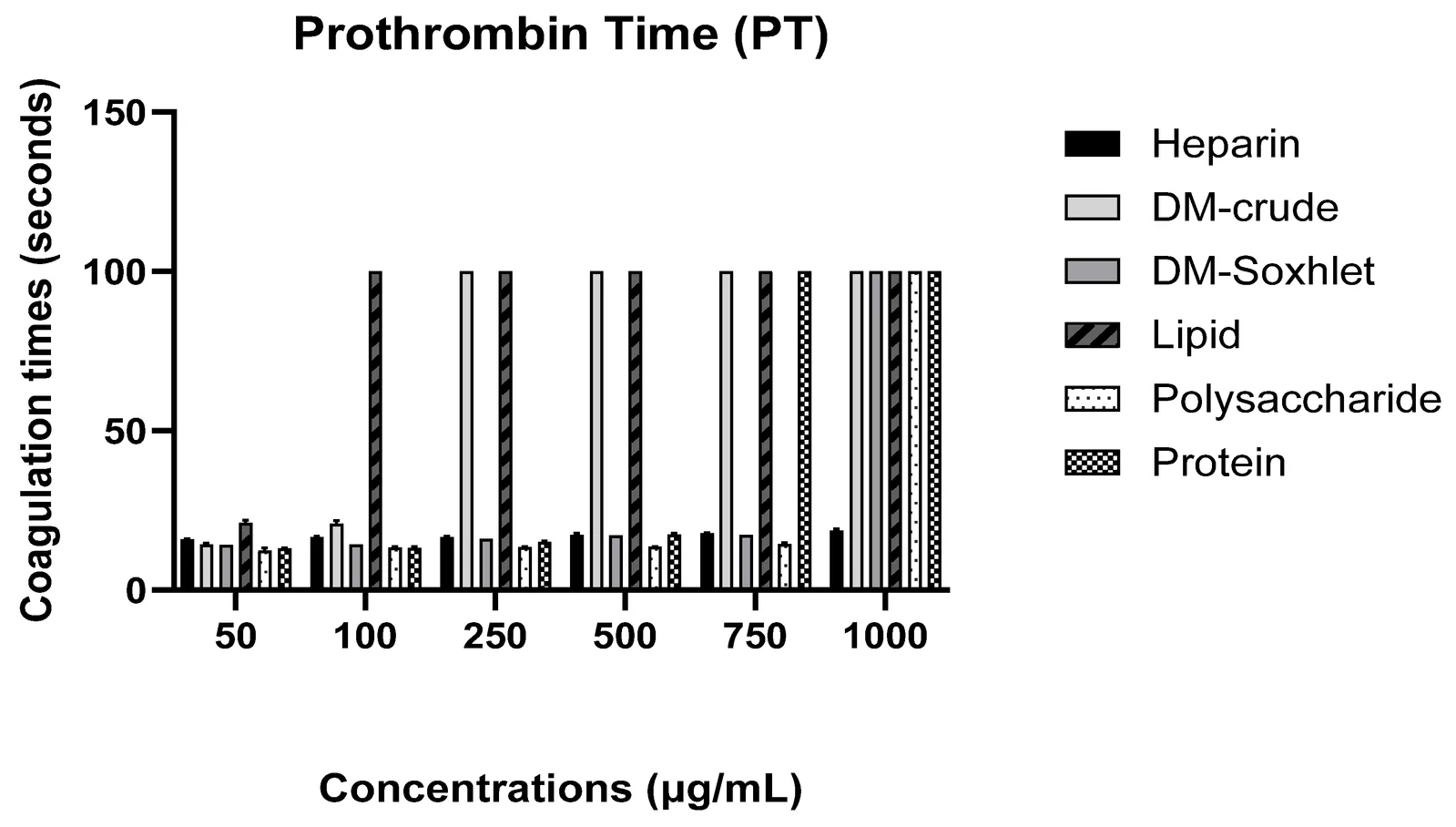
Effect of different extracts and heparin as standard on prothrombin time (coagulation time in seconds). The comparison at each concentration was performed with respect to Heparin. Error bars represent standard deviations.

The findings showed a highly significant difference between different extracts and heparin (*p* < 0.0001). Moreover, different extracts at varying concentrations produced varied effects on PT coagulation time (*p* < 0.0001). Heparin showed modest, concentration-dependent prolongation of PT, increasing from 15.96 ± 0.15 s at 50 µg/mL to 18.69 ± 0.44 s at 1000 µg/mL. The Protein extract showed anticoagulant activity, with PT increasing from 13.1 ± 0.2 s at 50 µg/mL to 17.46 ± 0.32 s at 500 µg/mL and exceeding the assay limit (>100 s) at concentrations ≥750 µg/mL. The Polysaccharide extract produced a gradual increase, from 12.46 ± 0.66 s at 50 µg/mL to 14.46 ± 0.45 s at 750 µg/mL, before exceeding 100 s at 1000 µg/mL. Notably, the lipid extract showed a pronounced effect, extending PT to >100 s starting at 100 µg/mL and remaining consistent up to 1000 µg/mL. The DM-Soxhlet extract showed moderate activity at lower concentrations, 14.2 ± 0.22 s at 50 µg/mL, but exceeded 100 s at 1000 µg/mL. The DM-crude extract displayed superior activity compared to heparin, with PT rising from 14.3 ± 0.45 s at 50 µg/mL to beyond the assay limit >100 s starting at 250 µg/mL and maintained through 1000 µg/mL. Overall, all extracts produced a dose-dependent prolongation of PT, with values reaching the analyzer’s upper detection limit (>100 s) at high concentrations. Since this represents the maximum measurable clotting time, further quantitative comparison among extracts and with heparin was not possible once the threshold was exceeded (Figure 3, Table 5).

#### 2.5.2. Effect on Partial Thromboplastin Time

**Figure 4:**
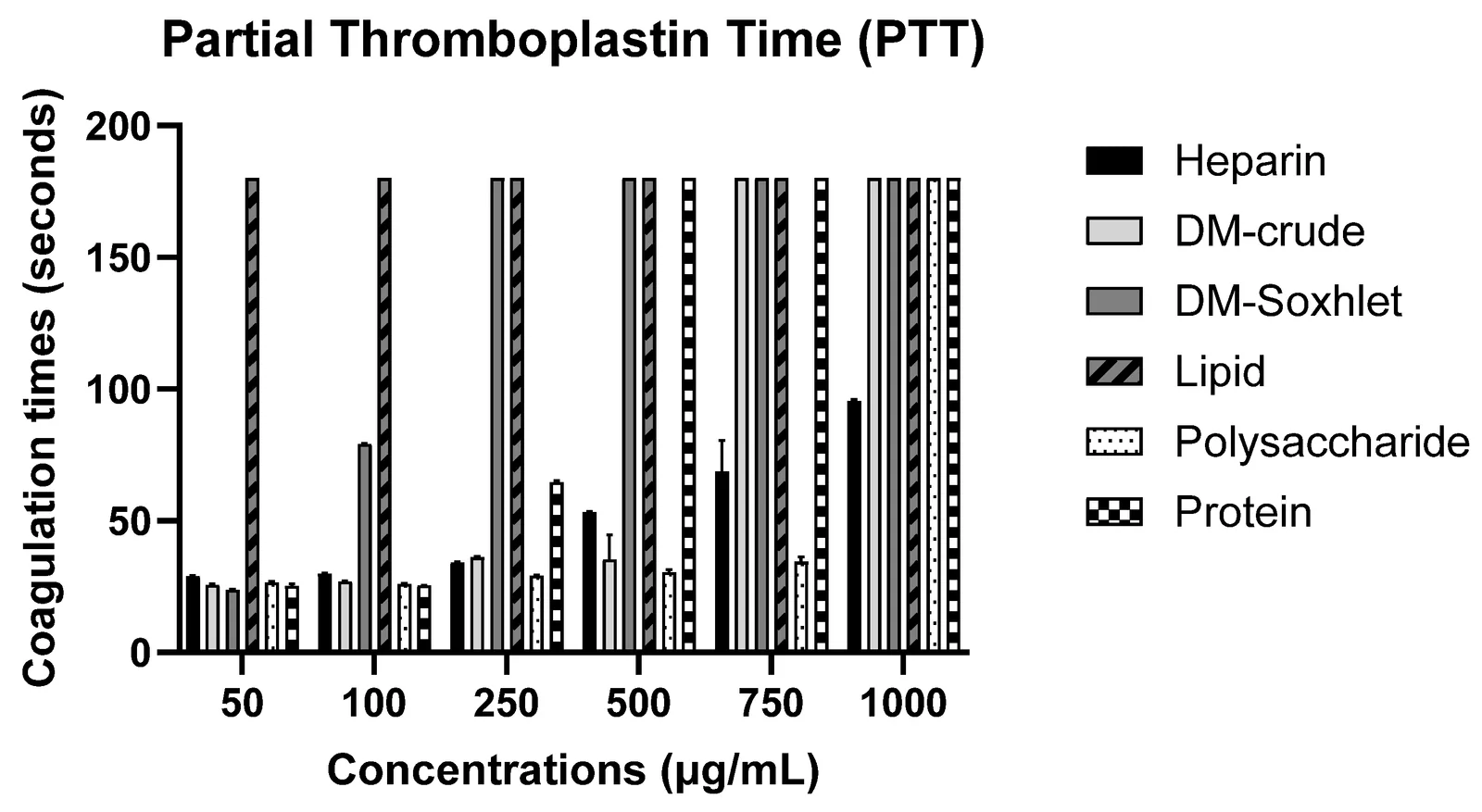
Effect of different extracts and heparin as standard on partial Thromboplastin time (coagulation time in seconds). The comparison at each concentration was performed in triplicate with respect to heparin. Error bars represent standard deviations.

Different extracts at varying concentrations exerted a significant effect on coagulation time (*p* < 0.0001). Heparin showed a clear concentration-dependent prolongation of PTT, increasing from 28.83 ± 0.40 s at 50 µg/mL to 95.46 ± 0.64 s at 1000 µg/mL. The protein extracts increased PTT values from 25.2 ± 0.81 s at 50 µg/mL to 64.5 ± 0.72 s at 250 µg/mL, exceeding the assay limit (>180 s) at concentration of 500 µg/mL. The polysaccharide extract showed a gradual increase, from 26.53 ± 0.45 s at 50 µg/mL to 34.36 ± 1.87 s at 750 µg/mL, with values exceeding >180 s at 1000 µg/mL. The lipid extracts prolonged PTT beyond the assay limit >180 s across all tested concentrations (50–1000 µg/mL). The DM-Soxhlet extract increased PTT to 79 ± 0.20 s at 100 µg/mL, with values exceeding >180 s from 250 µg/mL onward. The DM-crude extract showed a concentration-dependent increase from 25.56 ± 0.51 s at 50 µg/mL to 36.1 ± 0.26 s at 250 µg/mL, then plateauing at >180 s at 750 µg/mL and 1000 µg/mL. Overall, all extracts prolonged PTT in a dose-dependent manner (Figure 4, Table 6). However, because values exceeding 180 s represent the upper detection limit of the analyzer, further quantitative discrimination among the most active extracts was not possible once this limit was reached.

### 2.6. Results of Anti-Hemolytic Activity

**Figure 5:**
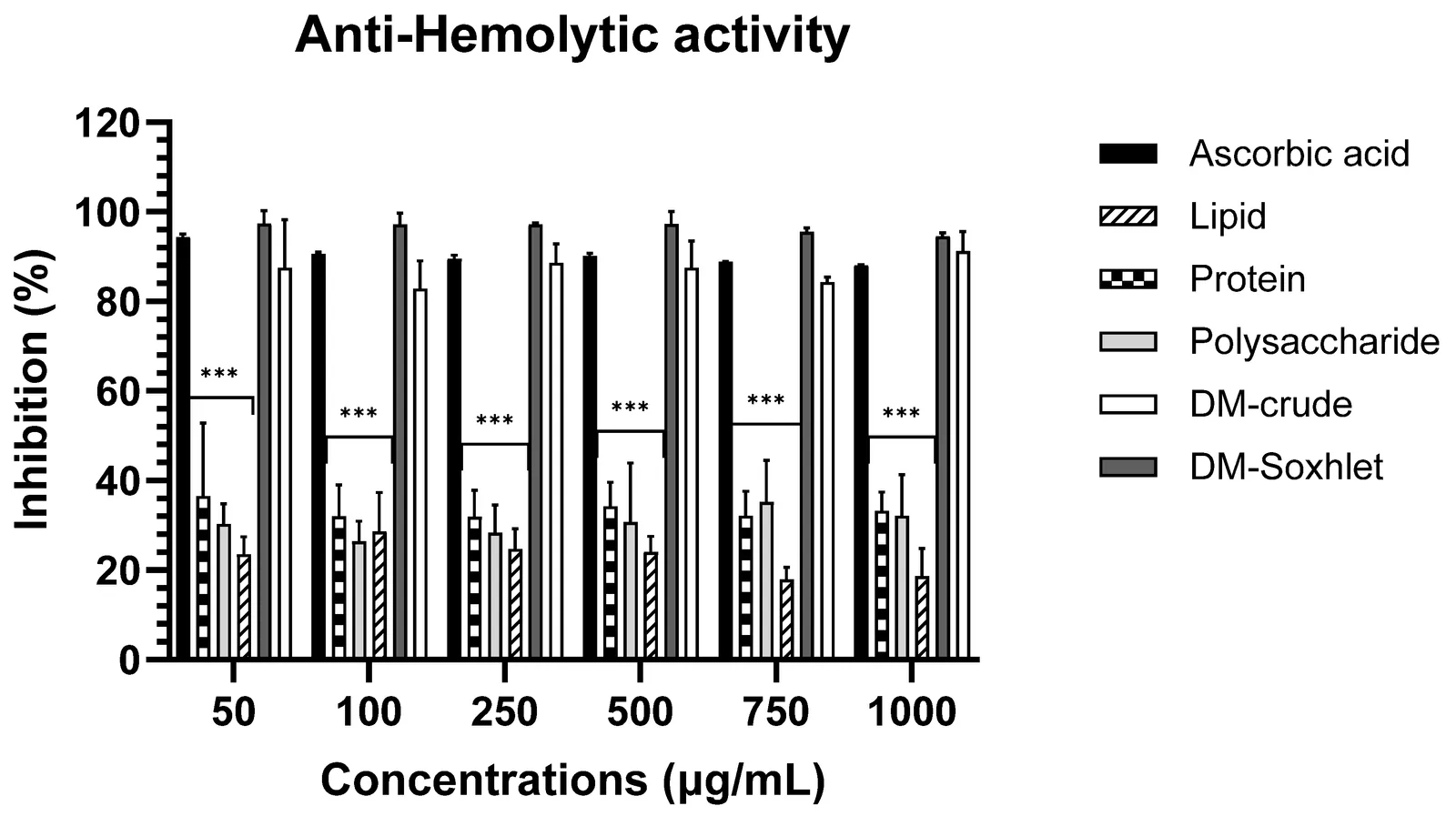
Percent inhibition of RBC hemolysis by various extracts and ascorbic acid as standard. The comparison at each concentration was performed with respect to ascorbic acid. Statistical significance is indicated as *p* < 0.001 (***). Error bars represent standard deviations.

The Dichloromethane Methanol crude extract and the Dichloromethane Methanol Soxhlet extract demonstrated a significant anti-hemolytic effect compared to ascorbic acid (Figure 5, Table 7). Notably, the concentration of the extracts did not significantly influence this effect (*p* > 0.05). At the lowest concentration tested, ascorbic acid exhibited a maximum inhibition of 94.35% at 50 µg/mL. Similarly, the DM-Soxhlet extract showed a high inhibition of 97.40% at the same lowest concentration, with no significant difference from ascorbic acid. The DM-crude extract demonstrated an inhibition of 87.51% at 50 µg/mL, also indicating no significant difference. In contrast, the protein, polysaccharide, and lipid extracts showed substantially weaker anti-hemolytic activity, with *p*-values smaller than 0.001, indicating that their effects differed significantly from ascorbic acid (Figure 5, Table 7).

### 2.7. Results of Anti-Inflammatory Activity

**Figure 6:**
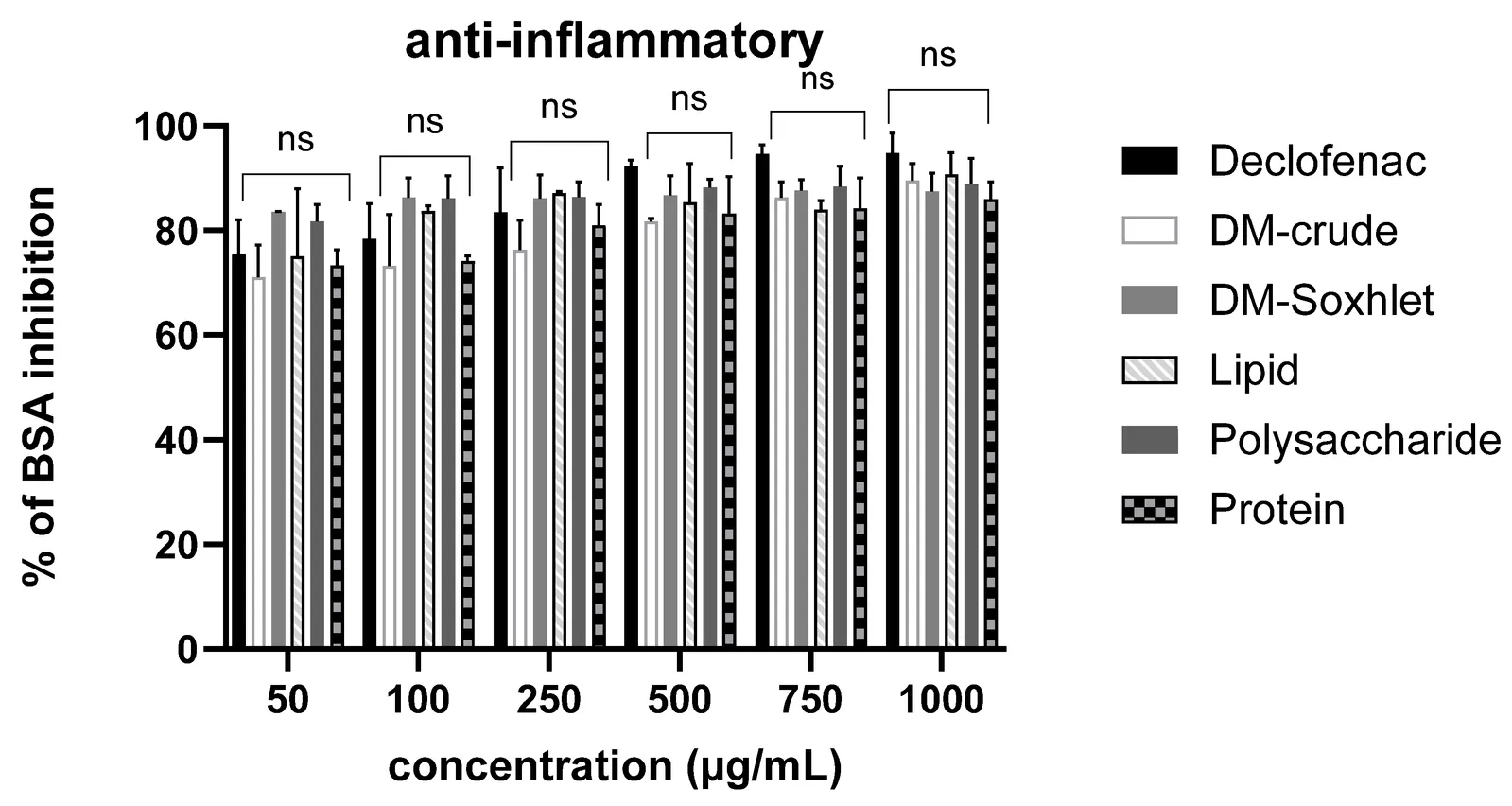
Percent inhibition of protein denaturation by various extracts and using diclofenac as standard. The comparison at each concentration was performed with respect to diclofenac. ns = not significant; *p* > 0.05. Error bars represent standard deviations.

All extracts tested showed significant inhibition of heat-induced denaturation of bovine serum albumin (BSA) (*p* < 0.0002), with activity comparable to the diclofenac sodium standard (Figure 6, Table 8). Importantly, inhibition was already pronounced at the lowest concentration tested (50 µg/mL), and although the response increased with dose, the rise was only slight and not strongly dose-dependent. Diclofenac exhibited 75.52 ± 6.52% inhibition at 50 µg/mL, reaching 94.81 ± 3.77% at 1000 µg/mL. The Protein extract showed 73.30 ± 2.99% inhibition at 50 µg/mL and 85.98 ± 3.33% at 1000 µg/mL. The Polysaccharide extract achieved 81.71 ± 3.20% inhibition at 50 µg/mL and 88.85 ± 4.92% at 1000 µg/mL. The Lipid extract displayed 75.02 ± 2.96% inhibition at 50 µg/mL and 90.71 ± 4.14% at 1000 µg/mL, representing one of the strongest effects at the highest concentration. The DM-Soxhlet extract showed 83.56 ± 0.05% inhibition at 50 µg/mL and 87.45 ± 3.48% at 1000 µg/mL. Finally, the DM-crude extract exhibited 71.04 ± 6.19% inhibition at 50 µg/mL and 89.54 ± 3.21% at 1000 µg/mL. Overall, all extracts demonstrated anti-inflammatory activity equivalent to diclofenac, achieving high levels of inhibition of heat-induced BSA denaturation across the tested concentrations, with only a modest increase in inhibition as the dose was raised (Figure 6, Table 8).

## 3. Discussion

Marine macroalgae, including Jania rubens, are increasingly recognized as rich sources of bioactive compounds with promising applications in pharmaceuticals and cosmetics industries. Despite Lebanon’s rich marine biodiversity, its algal resources remain relatively underexplored [35]. Therefore, this study aimed to investigate the chemical characterization of various extracts obtained from Lebanese *J. rubens*, along with their in vitro anti-diabetic, anti-coagulant, anti-inflammatory, and anti-hemolytic activities.

The inhibition of α-amylase, a first-line enzyme in carbohydrate digestion, delays the hydrolysis of polysaccharides, thereby reducing postprandial blood glucose levels, making it an effective approach for treating diabetes mellitus [36,37]. Many studies have reported the antidiabetic effects of marine algae, particularly red algae, which contain natural alpha-glucosidase inhibitors that can help reduce glucose absorption [38]. Previous studies highlighted the anti-diabetic and anti-obesity properties of *J. rubens* polyphenolic extracts [7] and the α-amylase inhibitory activity of methanol extracts [39]. Our results revealed that dichloromethane/methanol (DM-Soxhlet) and lipid extracts exhibited strong α-amylase inhibitory activity. GC–MS analysis of lipid extract tentatively identified bioactive compounds such as 2,4-Di-tert-butylphenol (a phenolic antioxidant), along with fatty acids including octadecanoic acid and hexadecenoic acid, which may contribute to the observed inhibitory activity through binding to α-glucosidase and α-amylase enzymes [7]. Furthermore, the DM-Soxhlet was shown to be rich in flavonoid derivatives [34], which may enhance insulin sensitivity and secretion, thereby contributing to glucose control and protecting pancreatic beta cells [40]. Notably, polysaccharide extracts also showed superior inhibition at high concentrations (500–750 and 1000 µg/mL). Our previous analysis revealed the presence of carrageenan and sulfated polysaccharide [25], compounds that enhance insulin sensitivity, promote glucose uptake, and lower blood glucose levels [41]. However, the protein extract exhibited weaker α-amylase inhibition compared to acarbose (IC_50_ 112 µg/mL vs. 71.6 µg/mL, *p* < 0.0001), likely due to the complex composition of the extract in which potential inhibitory peptides are present at lower concentrations and act through less specific mechanisms than the synthetic drug [42]. Overall, the DM-Soxhlet, lipid and polysaccharide extracts have potential as candidates for natural therapeutic interventions in diabetes management.

The search for novel anticoagulant agents from natural sources has gained considerable interest due to the limitations and side effects associated with conventional pharmaceuticals [43]. This study investigated the anticoagulant activity of different extracts of Lebanese *J. rubens* for the first time. Our data indicate that all tested extracts of *J. rubens* at varying concentrations (50–1000 µg/mL) exhibited inhibitory effects on both the extrinsic and intrinsic coagulation pathways, with clotting times comparable to heparin. At higher concentrations, several extracts reached the analyzer’s upper detection limits (>100 s for PT and >180 s for PTT), beyond which further quantitative comparison with heparin was not possible. Our findings are consistent with previous reports on red algae, where anticoagulant properties are often attributed to sulfated polysaccharides such as carrageenans or sulfated galactans [44]. The polysaccharides in *J. rubens* resemble typical carrageenan profiles, containing a high sulfate content (38%). The anticoagulant activity of sulfated polysaccharides is closely associated with their structural features, particularly glycosidic linkages, molecular weight, and characteristic sulfation patterns [45,46]. This effect is partly explained by electrostatic interaction between the negatively charged sulfate groups of these polysaccharides and the positively charged peptide sequences [47]. Carrageenan isolated from the red algae *Corallina* has also shown marked anticoagulant effects [35], with λ-carrageenan, in particular, exhibiting stronger activity than κ-carrageenan, due to its higher degree of sulfation [12,48]. Beyond polysaccharides, the protein extract of *J. rubens* demonstrated notable anticoagulant effects. Aligning with the increasing attention to anticoagulant peptides derived from marine sources [49]. Peptide sequence analysis identified 11 amino acids, including glutamic acid, valine, methionine, glycine, ornithine, and arginine, which are known to exert antiplatelet and anticoagulant activities [50,51,52]. In addition, Leucine-containing peptides have been linked to enhanced fibrinolytic and anticoagulant activity [53]. The lipid extract showed a marked anticoagulant effect; PT values exceeded the upper measurement limit of the analyzer (>100 s) from 100 µg/mL onward, while PTT values exceeded 180 s at all tested concentrations. GC-MS analysis revealed that the lipid extract is rich in stearic acid (Octadecanoic acid), which has been associated with improved stability of thrombin inhibitors such as hirudin, resulting in superior in vivo anticoagulant activity and prolonged half-life [54]. In summary, our investigation highlights that all *J. rubens* extracts demonstrated strong anticoagulant activity under the experimental conditions employed, however, because the PT and PTT values reached the upper detection limits of the analyzer, quantitative discrimination among the most active extracts and direct comparisons with heparin could not be reliably established. Therefore, the results should be interpreted as evidence of substantial anticoagulant potential rather than conclusive evidence regarding the underlying mechanisms or relative potency of the extracts. Future studies should employ more specific assays such as thrombin time, factor Xa inhibition, platelet aggregation, or antithrombin-mediated pathways and in vivo validation to confirm the precise mechanisms underlying the observed effects.

Inflammation is closely linked to protein denaturation, which results in the loss of biological functions and the release of pro-inflammatory mediators. This denaturation can be induced by external stress, such as heat, or by exposure to chemicals, such as organic solvents [55]. Algae extracts represent a promising alternative to non-steroidal anti-inflammatory drugs (NSAIDs), whose prolonged use may cause adverse effects on the gastrointestinal tract, kidneys, and liver [15]. Bioactive compounds present in algae, including phycocyanin, polysaccharides, fatty acids, terpenoids, and polyphenols, may modulate inflammatory pathways with fewer side effects [56]. Previous studies demonstrated that crude methanolic extract of *J. rubens* showed significant in vivo anti-inflammatory and hepatoprotective effects [57]. Our results further demonstrate that *J. rubens* exhibits in vitro activity in the BSA denaturation assay, equivalent to that of the standard drug diclofenac. GC-MS analysis tentatively identified various bioactive compounds in *J. rubens*, including 7-Methoxyflavone, pyrazole and benzopyran derivatives, all of which have shown anti-inflammatory properties [34]. The DM-crude also contained diterpenoids, terpenoids, saturated fatty acids and sterol ester compounds, which support its anti-inflammatory activity [58]. Polyphenolic compounds from *J. rubens* have been reported to contribute to these effects [7]. In addition, MAPK-like proteins (p38 and JNK homologues) have been detected in *J. rubens*, with phosphorylation peaking during low tide stress and subsequently decreasing, indicating transient activation [59]. Since MAPK pathways are known to regulate inflammatory responses through the modulation of pro-inflammatory mediators such as TNF-α, IL-1β, and IL-6 [60], it is possible that compounds present in *J. rubens* may influence inflammatory processes through related mechanisms. Similarly, IRAK activation through TLR and IL-1R signaling in microglia further amplifies NF-κB/MAPK pathways, leading to neuroinflammation and cytokine release [61]. However, these molecular pathways were not directly investigated in the present study, and their involvement in the biological effects observed here remains hypothetical and requires further experimental validation. Additionally, sulfated polysaccharide isolated from the red alga *Gracilaria cornea*, have demonstrated anti-inflammatory effects by inhibiting histamine release, neutrophil migration, and vascular permeability [62]. Similarly, carrageenans from red algae species have been recognized as anti-inflammatory agents in experimental animals [46]. Many anti-inflammatory bioactive peptides contain negatively charged amino acids such as aspartate and glutamate [63]. Sequences rich in hydrophobic residues (valine, isoleucine, proline) and positively charged amino acids (histidine, arginine, lysine) have been linked to anti-inflammatory responses [64]. In summary, the diverse chemical composition of *J. rubens* including bioactive compounds, flavonoids, aldehydes, quinolones, pyrazole, sulfated polysaccharide, fatty acids and essential amino acids demonstrate promising anti-inflammatory activity in vitro which highlight the importance of further molecular studies to elucidate the precise mechanisms by which *J. rubens* modulates inflammatory pathways.

Furthermore, exposure of red blood cells to various substances can lead to membrane lysis and hemoglobin oxidation, which are critical indicators of erythrocyte cytotoxicity [39]. Because the human red blood cell membrane has a component similar to that of lysosomal membrane [65], membrane stabilization is considered important in limiting the leakage of serum fluid and proteins into tissues during periods of increased vascular permeability triggered by inflammatory mediators [66]. Our results indicate the DM-crude and DM-Soxhlet extracts exhibit significant anti-hemolysis effect compared to ascorbic acid, achieving up to 90% inhibition at high concentration. In contrast, the protein, polysaccharide, and lipid extracts displayed relatively weak anti-hemolytic activity. Consistent with our results, a previous study demonstrated that the methanolic crude extract of *J. rubens* produced the strongest anti-hemolytic effect in a concentration-dependent manner when comparing the extract’s ability to interact with erythrocyte membranes, compared with diclofenac potassium. The authors suggest that this activity was attributed to potentially altering cell surface charges [67]. Moreover, silver nanoparticles synthesized from *J. rubens* have demonstrated significant anti-hemolytic and nephroprotective effects in animal studies against gentamicin-induced nephrotoxicity. This biological activity has been attributed to the presence of bioactive phytochemicals in the alga, including phenols, flavonoids, saponins, tannins, and alkaloids, which can stabilize erythrocyte membranes and modulate cellular responses [67].

In conclusion, the anti-hemolytic activity of *J. rubens* appears to be mediated by a diverse array of bioactive constituents, including phenolic compounds, flavonoids, saponins, tannins, alkaloids, and nanoparticles derived from the alga. It is important to note that while natural products are being investigated as alternative sources of bioactive compounds, their safety cannot be assumed without proper toxicological validation. These compounds contribute to erythrocyte membrane stabilization, protection against hypotonic stress, and modulation of cellular responses. Collectively, our findings, together with previous reports, highlight *J. rubens* as a promising natural source of anti-hemolytic agents with potential therapeutic applications.

## 4. Materials and Methods

### 4.1. Collection of Macroalgae

*J. rubens* algae used in this study were collected from the Mediterranean coast of northern Lebanon, specifically from Qalamoun, at a depth of 2–3 m in October 2023. Specific permission for the collection of this alga was obtained from the President of the Fishermen’s Cooperative in Qalamoun. The samples were identified by Professor Dr Hiba Mawlawi and confirmed by Professor Dr. Hussein Kannan, referring to the book of Belous and Kanaan [68]. After collection, the samples were rinsed with running water to eliminate debris and epiphytes, followed by washing with distilled water. After that, they were air-dried at room temperature in the dark for 5–7 days before being ground into a fine powder. A herbarium voucher for *J. rubens* (AZM-1105) is maintained at the Doctoral School of Science and Technology at the Lebanese University.

### 4.2. Extraction of Natural Compounds from J. rubens

#### 4.2.1. Dichloromethane/Methanol Soxhlet Extract

Forty grams of crushed *J. rubens* were placed in a Soxhlet apparatus for extraction. The process involved repeated washing with a dichloromethane: methanol (DM:M) (Fluka, Darmstadt, Germany) mixture in a 1:1 ratio conducted over an extended period ranging from 6 to 24 h. This method yielded 400 mg of DM-Soxhlet extract (DM-Soxhlet). The Final extract was evaporated using a rotary evaporator and stored in dark at −4 °C for later use.

#### 4.2.2. Dichloromethane/Methanol Crude Extract

Twenty-five grams of crushed *J. rubens* were extracted with 200 mL of (DM:M) ratio (1:1). The mixture was agitated for three consecutive days. Afterward, it was evaporated using a vacuum evaporation system, yielding 400 mg of crude extract (DM-crude). The Final extract was stored in the dark at −4 °C until subsequent use.

### 4.3. Fractionation Technique

The fractionation and extraction procedures were performed according to the method described in our previous work [25]. The algal biomass was sequentially fractionated to obtain the lipid, protein, and polysaccharide fractions as described below.

#### 4.3.1. Lipid Extraction

Thirty grams of dried, powdered red algae were extracted with a 400 mL mixture of Chloroform–Hexane solvent (2:1) ratio (Fluka, Darmstadt, Germany). The mixture was left under agitation overnight at room temperature. The following day, it was filtered, and the resulting supernatant was evaporated using a vacuum evaporation system, resulting in 140 mg of lipid extract. The remaining residue was subsequently kept for protein extraction.

#### 4.3.2. Protein Extraction

Two hundred millilitres of distilled water (ddH_2_O) preheated to 40 °C were added to the residues remaining after lipid extraction. The suspension was maintained under stirring for 24 h at the same temperature. It was then filtered, supplemented with additional 40 mL of distilled water, and filtered again. To precipitate proteins, 2 mL of barium chloride (1 M) and 2 mL of barium hydroxide (1 M) (Sigma-Aldrich, St. Louis, MO, USA) were gradually added while stirring at 60 °C. The mixture was subsequently centrifuged at 5000 rpm for 10 min at 4 °C. The pellet obtained was lyophilized, producing 113 mg of protein, whereas the supernatant was retained for polysaccharide extraction.

#### 4.3.3. Polysaccharide Extraction

The supernatant collected from the preceding step was filtered to eliminate any residual protein traces. Subsequently, 100 mL of ddH_2_O was added to the filtrate. The mixture was then centrifuged twice at 5000 rpm for 10 min at 4 °C. Finally, the recovered supernatant was lyophilized, resulting in 1.5 g of polysaccharide extract.

### 4.4. Characterization of J. rubens Extracts

The chemical constituents of *J. rubens* extracts were characterized using GC–MS, high-performance liquid chromatography (HPLC), and Fourier transform infrared (FTIR) spectroscopy.

#### 4.4.1. Gas Chromatography Mass Spectrometry Analysis (GC-MS)

GC-MS analysis of lipid and crude extracts of *J. rubens* was carried out using an Agilent Technologies GC system coupled with a mass selective detector (HP-5MS). Separation was achieved on a 5% phenyl methyl siloxane capillary column (30.0 m × 250 μm × 0.25 μm), with helium as the carrier gas at flow rate of 1 mL/min. The oven temperature was programmed to start at 90 °C for 1 min, then increase at 8 °C/min to 205 °C, followed by 5 °C/min to 240 °C, and finally 8 °C/min to 300 °C. The mass spectrometer was operated at 70 eV. Compound identification was based on comparison of the obtained mass spectra data with reference spectra from the National Institute of Standards and Technology (NIST). Although authentic standards were not available due to resource limitations, high NIST match percentages were used to support metabolite identification, providing reliable preliminary characterization of the extracts.

#### 4.4.2. High-Performance Liquid Chromatography (HPLC)

Protein amino acid characterization was performed using an Agilent 6490 Triple Quadrupole LC/MS system equipped with an electrospray ionization (ESI) source and operated with Agilent MassHunter Acquisition software, following the LC-MS/MS approach described by George et al. (2010) [69], with minor modifications. Identification and quantification were carried out using amino acid standards (glutamic acid, aspartic acid, tyrosine, citrulline, arginine, phenylalanine, methionine, ornithine, leucine, valine, alanine, and glycine) (Table S1). Chromatographic separation was achieved on a Chromolith RP-18 column (100 × 4.6 mm, 5 µm) maintained at 40 °C, using a mobile phase composition of water containing 0.1% formic acid (20%) and methanol containing 0.1% formic acid (80%), with a flow rate of 0.500 mL/min. Samples were injected under standard injection conditions using an injection volume of 5.0 µL, and detection was performed in positive ion mode (ESI+) using MRM acquisition. Calibration was performed using a stock amino acid standard solution (5000 mg/L), and working solutions were prepared by serial dilution to obtain 10–1000 mg/L (prepared in methanol and stored at 4 °C until analysis). The amino acid analysis in the present study was conducted primarily to compare the amino acid profiles among our samples rather than to establish a fully validated analytical quantitative method [69].

### 4.5. Investigation of Biological Activities

#### 4.5.1. Institutional Review Board-Approved Participant Recruitment

Participants were enrolled in the study in accordance with the most recent Declaration of Helsinki guidelines for research Involving Human Subjects. Ethical approval was granted by the Clinical Research Ethics Committee of the Institutional Review Board (IRB) at Lebanese University (IRB code CE-EDST-4-2025). Prior to participation, each individual completed the consent procedure, which included signing a consent form and completing a questionnaire.

#### 4.5.2. In Vitro Test on the Activity of α-Amylase

The inhibition of α-amylase was assessed using the starch-iodine [70]. In this assay, 40 μL of each sample, including acarbose (Thermo Scientific, Waltham, MA, USA) as the reference standard and extract concentrations of 50, 100, 250, 500, 750, and 1000 μg/mL, were combined with 40 μL of 1% α-amylase (Sigma-Aldrich, Darmstadt, Germany) prepared in phosphate-buffered saline (PBS) (20 mM, PH 6.9) (HiMedia Laboratories Pvt. Ltd., Mumbai, India). The reaction mixture was then incubated at 37 °C for 5 min. Following this, 40 μL of 1% starch (prepared with boiled PBS 0.1 M) was added, and the mixture was incubated again at 37 °C for 25 min. Afterward, 80 μL of 0.1 M hydrochloric acid (HCl) (VWR, Radnor, PA, USA) and 40 μL of the 2.5 mM iodine solution (Sigma-Aldrich, St. Louis, MO, USA) were added. The negative control included all reagents (starch, HCL and iodine) but excluded the amylase, while the positive control contained all the reagents without the extract. Dimethyl sulfoxide (DMSO) (J.T.Baker, Radnor, PA, USA) served as the Blank. Absorbance readings were performed in triplicate using a 96-well microtiter plate and an ELISA reader (biomérieux, Lyon, France) at a wavelength of 630 nm. The IC_50_ value, defined as the concentration required to inhibit 50% of α-amylase activity, was determined from the graph plotting inhibition against extract concentration, and was compared with that of acarbose. The inhibition percentage was calculated using the following equation:

Inhibition (%) = (1 − (OD Test sample/OD negative control)) × 100.

#### 4.5.3. In Vitro Anticoagulant Activity

In vitro anticoagulant activity was evaluated according to the method reported by Hmidani A et al. [71] with slight modification to suit the specific kit employed and analyzer used. Blood samples were obtained from three healthy volunteers who were not receiving any medications and who had normal prothrombin time (PT) and activated partial thromboplastin time (APTT) values. The blood was collected in citrated tubes, and centrifuged at 3000 rpm for 10 min to separate the citrated plasma. All PT and aPTT assays were performed using a CL analyzer (China).

For the APTT assay, 100 μL of normal citrated plasma sample was mixed with 50 μL of extract at different concentrations 50, 100, 250, 500, 750, and 1000 μg/mL for 10 min at 37 °C according to the manufacturer’s instructions. After this step, 100 μL of APTT reagent (from Hemosil synthasil PTT kit, Bedford, MA, USA) was added and incubated for an additional 3 min at 37 °C. After that, 100 μL of 0.25 M CaCl_2_ (from Hemosil synthasil PTT kit, Bedford, MA, USA) was added to the mixtures and the clotting time was recorded using a CL analyzer [72].

For the PT assays, 100 μL of normal citrated plasma was incubated with 50 μL of each extract at varying concentrations of 50, 100, 250, 500, 750, and 1000 μg/mL for 10 min at 37 °C according to the manufacturer’s instructions. Clotting time was then measured immediately after the addition of 100 μL of PT reagent (from Hemosil, RecombiPlasTin^®^ 2G PT kit, Bedford, MA, USA) [72]. Heparin sodium (25,000 I.U/5 mL) was used as the reference standard for PT and aPTT assays prepared at the same concentration as the extracts. Normal plasma without the extract served as the control. Experiments were performed in triplicate to ensure reproducibility.

#### 4.5.4. In Vitro Anti-Inflammatory Activity

The anti-inflammatory potential of different extracts was assessed by the albumin denaturation inhibition assay with slight modifications [55]. The reaction mixture included 2 mL of 1% bovine serum albumin (BSA) (Sigma-Aldrich, St. Louis, MO, USA) with 400 μL of extracts, including diclofenac sodium as a standard at concentrations of 50, 100, 250, 500, 750, and 1000 μg/mL. The reaction mixture was first incubated at 37 °C for 20 min, and then heated at 70 °C for 10 min. After allowing it to cool, turbidity was determined in triplicates using an ELISA reader (biomérieux, Lyon, France) at a wavelength of 660 nm. For the negative control, an equal volume of DMSO replaced the extract, while the PBS served as the blank. The IC_50_ value, defined as the concentration of the sample needed to inhibit 50% of protein denaturation, was determined from the graph plot of percentage inhibition versus extract concentration, and compared with the IC_50_ of diclofenac sodium. The percentage inhibition of protein denaturation was calculated using the following equation:

Anti-denaturation Activity (%): (1 − (OD Test sample/OD negative control)) × 100

#### 4.5.5. In Vitro Anti-Hemolytic Activity

The anti-hemolytic activity of various extracts was assessed by a spectrophotometric method previously described [35]. Briefly, ten milliliters of EDTA-anticoagulated blood was collected from three healthy persons with no known blood-related disorders and centrifuged at 3000 rpm for 10 min. The supernatant was removed, and the remaining erythrocyte pellet was washed three times with 0.2 M PBS (pH 7.4), followed by another centrifugation for 10 min at 3000 rpm. The final pellet was then resuspended in 10 mL of saline solution (0.9% NaCl). Next, 0.4 mL of ascorbic acid (Sigma-Aldrich, St. Louis, MO, USA) as a standard and extracts at concentrations of 50, 100, 250, 500, 750 and 1000 μg/mL were mixed with 0.4 mL of erythrocyte suspension and incubated at 37 °C for 5 min. Oxidative damage to membrane lipids was induced by adding 0.2 mL of H_2_O_2_ (0.82 M in PBS), followed by incubation at 37 °C for 3 h with shaking at 120 rpm. After incubation, the samples were centrifuged at 3000 rpm for 10 min, and the absorbance of the supernatant was recorded in triplicate at 540 nm. The negative control consisted of the erythrocyte suspension without H_2_O_2_, while 0.2 M PBS was used as the blank. The IC_50_ value, defined as the concentration required to inhibit 50% of RBC hemolysis, was calculated from the graph plot of percentage of inhibition versus extract concentration and compared to the IC_50_ of the standard ascorbic acid. The percentage of inhibition was calculated using the following equation:

Inhibition (%): (1 − (OD Test sample/OD negative control)) × 100.

### 4.6. Statistical Analysis

In the current study, one-way ANOVA, two-way ANOVA, multiple comparisons, and non-linear regression fitting models were performed. A two-way ANOVA was consistently applied to evaluate the effects of extract type, concentration, and their interaction on the observed biological activities. Post hoc multiple comparison analyses (Tukey’s test) were subsequently conducted to identify significant differences among extracts and concentrations where appropriate. GraphPad Prism v10.2.0 software was used for all statistical analyses. All experiments were conducted in triplicate, and the results are presented as means ± standard deviation (SD), obtained from three technical (measurement) replicates performed on a single extraction batch of algal material. Statistical significance was expressed as follows: *p* < 0.05 (*), *p* < 0.001 (**), *p* < 0.0001 (***), *p* < 0.00001 (****).

## 5. Conclusions

The Lebanese *J. rubens* extracts are rich in bioactive compounds, including proteins, lipids, flavonoids, and polysaccharides, demonstrating promising in vitro anti-diabetic, anti-coagulant, and anti-inflammatory activity with anti-hemolytic properties. The observed α-amylase inhibition, modulation of coagulation pathways, and anti-inflammatory activity with protection against hemolysis highlight the preliminary bioactive potential of these extracts. Future studies should aim to isolate and identify the bioactive compounds responsible for these effects, elucidating their mechanisms of action, and conducting in vivo validation and toxicity assessments to establish efficacy and safety.

## Figures and Tables

**Table 1:** Amino acid composition of *J. rubens* protein extract by HPLC-MS.

Amino Acid	R.T (min)	Precursor Ion	Product Ion	Dwell	Fragment (V)	Collision Energy (V)	Relative Abundance (%)
Glutamic acid	1.263	260.2	158.1	200	100	9	24.6
Aspartic acid	1.266	246.2	144.1	200	100	9	18.2
Leucine	1.29	188.1	86	200	100	9	12.4
Tyrosine	1.27	238.2	136.1	200	100	9	9.8
Arginine	1.277	231.2	70.1	200	100	9	8.6
Phenylalanine	1.28	222.2	120.1	200	100	9	7.9
Methionine	1.283	206.2	104.1	200	100	9	6.4
Ornithine	1.287	189.2	70.1	200	100	14	6
Valine	1.294	174.2	72.1	200	100	9	6
Alanine	1.297	146.1	44	200	100	9	4.8
Glycine	1.3	132.1	76.1	200	100	9	5.1

The table presents R.T: retention times (min), precursor ions, product ions, dwell times, fragment voltages, collision energies and relative abundance for each id.

**Table 2:** GC-MS profile of lipid extract.

Name of the Compound	Chemical Class	R.T (min)	*m*/*z* Value	% Area	M.w (g/mol)	Molecular Formula
1,8-Nonadien-3-ol	Unsaturated alcohol (allylic alcohol)	14.290	57	0.89	140.23	C_9_H_16_O
Dodecanoic acid	Saturated fatty acid	20.065	57	0.49	200.33	C_12_H_24_O_2_
Eicosanoic acid	Saturated fatty acid	29.138	57	0.73	312.53	C_20_H_40_O_2_
4-Trifluoroacetoxypentadecane	Alkane derivative	32.142	69.1	0.31	342.4	C_17_H_31_F_3_O_2_
2,4-Di-tert-butylphenol	Phenolic compound	36.013	191.1	2.48	206.32	C_14_H_22_O
2-Tetradecene	Unsaturated hydrocarbon	38.335	85.1	0.62	196.37	C_14_H_28_
1-Octadecene	Unsaturated hydrocarbon	40.503	57	0.35	252.48	C_18_H_36_
Neophytadiene	Diterpene hydrocarbon	44.807	105.1	0.19	278.52	C_20_H_38_
Octadecanoic acid	Saturated fatty acid	46.015	149	1.24	284.48	C_18_H_36_O_2_
hexadecanoic acid	Saturated fatty acid	48.879	97.1	0.35	256.42	C_16_H_32_O_2_
Phytol	Diterpene alcohol	52.038	85.1	0.61	296.54	C_20_H_40_O

The table lists the identified compounds, their chemical group, retention times (RT, min), mass-to-charge (*m*/*z*) values, relative percentage area (% area), molecular masses (M.w) (g/mol), and molecular formulas. RT refers to retention time in minutes, and m/z indicates the mass-to-charge ratio of the detected ions.

**Table 3:** Main compounds identified in *J. rubens* DM-crude extract by GC-MS.

Name of the Compound	Chemical Class	M.W (g/mol)	Molecular Formula	RT (min)	% Area	NIST Match (%)
Neophytadiene	diterpenoid	278.52	C_20_H_38_	45.179	8.24	91
2-Pentadecanone, 6,10,14-trimethyl-	terpenoids	268.5	C_18_H_36_O	45.349	2.56	86
Hexadecanoic acid	saturated fatty acids	256.42	C_16_H_32_O_2_	47.362	1.66	95
Phytol	terpenoids	296.53	C_20_H_40_O	51.604	2.98	93
Cholesteryl 3-cyclohexylbutyrate	sterol esters	538.90	C_37_H_62_O_2_	57.132	17.92	86

The table presents the chemical compound name, chemical classes, molecular weight (M.W) (g/mol), molecular formulas, retention times (RT, min), relative percentage areas (% area) and NIST match (%) of the detected compounds.

**Table 4:** Percent inhibition of α-amylase at different concentrations (50–1000 µg/mL) for various extracts and Acarbose as standard inhibitor.

	% Inhibition of α-Amylase					
	Concentration (µg/mL)					
	50	100	250	500	750	1000
Acarbose	65.08 ± 0.78	72.89 ± 3.17	72.15 ± 4.44	71.47 ± 5.42	71.59 ± 2.36	71.57 ± 4.19
Protein	47.64 ± 1.86	56.82 ± 2.68	64.36 ± 1.34	58.69 ± 2.78	56.81 ± 1.68	54.25 ± 2.20
Polysaccharide	46.41 ± 1.56	65.08 ± 0.95	68.86 ± 1.78	75.97 ± 2.02	75.21 ± 1.14	85.70 ± 3.57
Lipid	65.10 ± 3.36	70.14 ± 3.22	67.18 ± 1.66	70.67 ± 3.08	69.09 ± 0.59	69.90 ± 1.07
DM-Soxhlet	68.16 ± 4.13	69.27 ± 2.71	70.81 ± 5.31	58.14 ± 4.12	55.25 ± 1.61	50.99 ± 2.18
DM-crude	61.36 ± 1.69	59.89 ± 2.03	65.83 ± 1.63	54.76 ± 3.73	54.67 ± 2.52	59.52 ± 3.84

Data are expressed as mean ± SD from triplicate experiments. Statistical significance was determined relative to the control, with *p* < 0.05 considered significant.

**Table 5:** Coagulation time of prothrombin in seconds for various extracts and heparin as standard at different concentrations.

PT (Seconds)	50	100	250	500	750	1000
**Heparin**	15.96 ± 0.15	16.56 ± 0.41	16.53 ± 0.35	17.36 ± 0.61	17.80 ± 0.15	18.69 ± 0.44
**Protein**	13.10 ± 0.20 ****	13.16 ± 0.56 ****	15.16 ± 0.30 ***	17.46 ± 0.32 ns	100 ****	100 ****
**Polysaccharide**	12.46 ± 0.66 ****	13.26 ± 0.47 ****	13.43 ± 0.30 ****	13.60 ± 0.09 ****	14.46 ± 0.45 ****	100 ****
**Lipid**	21.00 ± 0.91 ****	100 ****	100 ****	100 ****	100 ****	100 ****
**DM-Soxhlet**	14.20 ± 0.22 **	14.30 ± 0.10 ****	16.00 ± 0.20 ns	17.20 ± 0.42 ns	17.30 ± 0.90 ns	100 ****
**DM-crude**	14.30 ± 0.45 ****	20.86 ± 1.00 ****	100 ****	100 ****	100 ****	100 ****

Data are expressed as mean ± standard deviation (SD) from triplicate experiments. **** *p* < 0.0001; *** *p* < 0.001; ** *p* < 0.01; ns: not significant *p* > 0.05. Values exceeding the analytical measuring range of the instrument were recorded as the maximum measurable value for statistical purposes (PT > 100 s reported as 100 s).

**Table 6:** Coagulation time of partial thromboplastin time in seconds for various extracts and heparin as standard at different concentrations.

PTT (Seconds)	50	100	250	500	750	1000
**Heparin**	28.83 ± 0.40	29.86 ± 0.15	34.06 ± 0.20	53.16 ± 0.30	68.53 ± 0.81	95.40 ± 0.64
**Protein**	25.2 ± 0.81 ns	25.33 ± 0.28 ns	64.5 ± 0.72 ****	180 ****	180 ****	180 ****
**Polysaccharide**	26.53 ± 0.45 ns	26.00 ± 0.15 ns	29.03 ± 0.45 ns	30.36 ± 1.00 ****	34.36 ± 1.87 ****	180 ****
**Lipid**	180 ****	180 ****	180 ****	180 ****	180 ****	180 ****
**DM-Soxhlet**	23.70 ± 0.26 ns	79.00 ± 0.20 ****	180 ****	180 ****	180 ****	180 ****
**DM-crude**	25.50 ± 0.51 ns	26.83 ± 0.25 ns	36.10 ± 0.26 ns	35.2 ± 9.45 ****	180 ****	180 ****

Data are expressed as mean ± standard deviation (SD) from triplicate experiments. Statistical significance is indicated as **** *p* < 0.0001; ns = not significant; *p* > 0.05. Values exceeding the analytical measuring range of the instrument were recorded as the maximum measurable value for statistical purposes (PTT > 180 s reported as 180 s).

**Table 7:** Percent inhibition of hemolysis for various extracts and ascorbic acid as control.

	Percent Inhibition of RBC Hemolysis					
	Concentration (µg/mL)					
	50	100	250	500	750	1000
**Ascorbic**	94.35 ± 0.68	90.65 ± 0.38	89.58 ± 0.72	90.10 ± 0.64	88.86 ± 0.04	88.03 ± 0.17
**Protein**	36.56 ± 6.24	32.02 ± 7.01	31.95 ± 5.88	34.21 ± 5.47	32.16 ± 5.46	33.22 ± 4.22
**Polysaccharide**	30.39 ± 4.43	26.42 ± 4.53	28.35 ± 6.17	30.77 ± 13.10	35.23 ± 9.32	32.17 ± 9.18
**Lipid**	23.54 ± 3.91	28.61 ± 8.75	24.78 ± 4.41	24.06 ± 3.46	17.95 ± 2.73	18.60 ± 6.22
**DM-Soxhlet**	97.40 ± 2.87	97.20 ± 2.52	97.15 ± 0.41	97.25 ± 2.83	95.51 ± 0.92	94.59 ± 0.77
**DM-crude**	87.51 ± 10.7	82.85 ± 6.18	88.67 ± 4.17	87.55 ± 6.85	84.38 ± 1.11	91.23 ± 4.34

Data are expressed as mean ± standard deviation (SD) from triplicate experiments. Values are presented as mean ± SD from three independent experiments. Statistical significance is indicated a with *p* < 0.05.

**Table 8:** Percent inhibition of protein denaturation by various extracts and diclofenac as control.

	% Inhibition of Heat-Induced BSA Denaturation					
	Concentration (µg/mL)					
	50	100	250	500	750	1000
**Diclofenac**	75.52 ± 6.52	78.38 ± 6.72	83.43 ± 8.54	92.29 ± 1.18	94.59 ± 1.82	94.81 ± 3.77
**Protein**	73.30 ± 2.99	74.14 ± 0.96	80.98 ± 4.00	83.21 ± 7.09	84.23 ± 5.82	85.98 ± 3.33
**Polysaccharide**	81.71 ± 3.20	86.14 ± 4.28	86.39 ± 2.88	88.24 ± 1.55	88.38 ± 3.90	88.85 ± 4.92
**Lipid**	75.02 ± 2.96	83.74 ± 0.95	87.09 ± 0.39	85.34 ± 7.42	83.94 ± 1.79	90.71 ± 4.14
**DM-Soxhlet**	83.56 ± 0.05	86.27 ± 3.77	86.12 ± 4.5	86.70 ± 3.76	87.66 ± 2.00	87.45 ± 3.48
**DM-crude**	71.04 ± 6.19	73.21 ± 9.82	76.32 ± 5.67	81.71 ± 0.6	86.27 ± 3.01	89.54 ± 3.21

Data are expressed as mean ± standard deviation (SD) from triplicate experiments. Values are presented as mean ± SD from three independent experiments.

## Data Availability

All data and materials support our published claims and comply with the field standards. The original data can be made available upon request.

## Supplementary Materials

The following supporting information can be downloaded at: https://www.mdpi.com/article/10.3390/md24090295/s1, Table S1: List of amino acid standards used for HPLC-MS analysis.

## Abbreviations

The following abbreviations are used in this manuscript:

*J. rubens*	*Jania rubens*
DM	Dichloromethane methanol
M	Methanol
