# Supplementary material for: Chemical Profiling and In Vitro Bioactivities of Extracts from the Red Alga Jania rubens Collected from the Lebanese Coast
Source: Mar Drugs. 2026 Aug 24;24(9):295. doi: 10.3390/md24090295 (PMC13608612; doi:10.3390/md24090295)
Supplement: Supplementary file 1 [file marinedrugs-24-00295-s001.zip › marinedrugs-4443005-supplementary.pdf]

Supplementary Data

Table S1. **List of Amino Acid Standards Used for HPLC-MS Analysis**

Amino Acid	Precursor Ion (m/z)	Product Ion (m/z)	Polarity
Glutamic acid	260.2	158.1	Positive
Aspartic acid	246.2	144.1	Positive
Tyrosine	238.2	136.1	Positive
Citrulline	232.2	13.1	Positive
Arginine	231.2	70.1	Positive
Phenylalanine	222.2	120.1	Positive
Methionine	206.2	104.1	Positive
Ornithine	189.2	70.1	Positive
Leucine	188.1	86	Positive
Valine	174.2	72.1	Positive
Alanine	146.1	44	Positive
Glycine	132.1	76.1	Positive
